# Tokenization efficiency of current foundational large language models for the Ukrainian language

**DOI:** 10.3389/frai.2025.1538165

**Published:** 2025-08-13

**Authors:** Daniil Maksymenko, Oleksii Turuta

**Affiliations:** ^1^Department of Artificial Intelligence, Kharkiv National University of Radio Electronics, Kharkiv, Ukraine; ^2^Computer Science and Artificial Intelligence Institution, V. N. Karazin Kharkiv National University, Kharkiv, Ukraine

**Keywords:** tokenization, large language model, corpus, domain, low-resource language

## Abstract

Foundational large language models (LLMs) are deployed in multilingual environments across a range of general and narrow task domains. These models generate text token by token, making them slower and more computationally expensive for low-resource languages that are underrepresented in the tokenizer vocabulary. It also makes their usage more costly in such cases, as pricing usually depends on the number of input and output tokens. This study compares multiple tokenizers of pretrained LLMs for the Ukrainian language. It also provides tokenization fertility measurements for current state-of-the-art (SOTA) models, both in terms of general-purpose language and specific domains, as well as results of experiments with a transliteration approach to make tokenization more efficient without information loss. The results provide insights into the current models’ disadvantages and possible problems in terms of Ukrainian language modeling.

## 1 Introduction

Tokenizers are an essential part of modern language models, as they both transform text data into a numerical format and split the text into smaller segments to reduce the dimensionality of inputs. Due to the inclusion of multilingual samples in pretraining datasets, recent large language models (LLMs) can execute tasks not only in their primary language (the dominant language of the training dataset in terms of the quantity of samples to represent this specific language) but also in others, even if those were not represented in large quantities during tuning (Erdem et al., 2022). For example, the Llama 3 training report states that 95% of the training dataset consisted of English and code, while only 5% represented other languages (Dubey et al., 2024). Nevertheless, this family of models can understand and generate text in other languages, such as Ukrainian. However, tokenizers for such models are not designed to process multilingual inputs and outputs equitably across all languages, especially low-resource ones (Ahia et al., 2023). At the same time, the quality and scope of the tokenizer vocabulary directly affect the accuracy and efficiency of the model built on top of it (Padalko et al., 2023).

The most common current approaches are Byte Pair Encoding (BPE) and SentencePiece-like (Gallé, 2019; Kudo and Richardson, 2018) tokenizers. Unlike older versions, they have fallback mechanisms in case of unknown tokens, so each word or even symbol would still receive a token set (whether it is a subword, character, or byte sequence). Thus, tokenizers should work even for low-resource languages, which do not get many subwords allocated in the vocabulary. However, it also leads to longer tokenized sequences (Limisiewicz et al., 2023). It was demonstrated earlier that the efficiency of LLM tokenization differs for each language, so they do not use the same number of tokens for the same information sample written in different languages (Petrov et al., 2023). Thus, the cost and speed of 100-character text generation in a primary and non-primary language of a tokenizer may differ considerably.

The majority of open-source or proprietary language models were trained primarily for English, so their tokenizers would segment English text better due to a larger number of tokens dedicated to English words (the example of Llama 3 was already mentioned earlier in the introduction). This can give the impression that tokenization is a solved problem for English, when in fact BPE tokenizers tend to have more tokens in the most used language of the dataset used to train them. If the training dataset is skewed toward Ukrainian, Georgian, or any other language, it would segment them better. The problem has been partially solved by extending the vocabulary size and training additional token embeddings.

Even switching between multiple domains in the same language can deteriorate the performance of the tokenizer as it has to split fewer common words, which are not covered as well as common ones in the vocabulary (Sachidananda et al., 2021). For example, the number of tokens to encode a medical text and an online blog on cinema may be significantly different, even if they have the same number of characters. Models and their tokenizers get biased toward the most frequent expressions they get in training datasets, so the performance for the same task (question answering, for example) can change due to the style and topic of the text. This would make the usage of foundational models even less efficient for domain-specific tasks in low-resource languages.

Tokenization efficiency can be defined as the number of tokens a tokenizer uses to represent the input text while preserving the integrity of the original text. Efficient tokenization reduces the computational cost of the causal language modeling task by minimizing the length of the token sequence without information loss or misinterpretation. Such tokenizers encode text in linguistically meaningful units and rarely turn to a character or byte fallback mechanism. They should be resistant to agglutination (addition of affixes should not lead to the tokenizer falling back to single characters or bytes) and other morphological transformations such as inflection (expression of tense, number, and gender), derivation (addition of an affix, which changes the grammatical category of a word), and others. As storing all possible word variations in the tokenizer vocabulary would be impossible due to the size of such a vocabulary and the constant change of natural spoken language, tokenizers should decompose forms into reusable morphemes. For example, the word “unhappy” should be tokenized as “un” and “happy,” which would ease the generalization of a language model as it learns a representation for frequent parts of a word instead of fragmented segments without a semantic meaning.

While SentencePiece and BPE tokenizers are trained to search for frequent character co-occurrences to determine the token content, they still encode some morphemes as separate tokens without mixing them or splitting them into sets of frequent character sequences. However, they are still morphologically unaware, which leads to token boundaries that do not correspond to stems or morphemes.

Such encoding makes natural language understanding (NLU) task learning more difficult and creates even more issues for languages with high morphological flexibility (Batsuren et al., 2024).

Morphological alignment of tokenizers is still an open area of research. Some experiments lead to conclusions that models based on BPE tokenizers achieve the same performance for NLU tasks as morphologically aligned tokenizers (Arnett et al., 2024), while others suggest the usage of tokenization schemes such as the unigram language model (ULM) (Kudo, 2018; Peters and Martins, 2022).

In the case of multilingual models, a tokenizer also requires consistency between all supported languages to be called efficient. This means it has to use a similar number of tokens to encode semantically identical texts in different languages. Tokenization efficiency directly influences the cost of inference, as users who request a generation in a non-primary language of the model would have to wait longer for the generation to complete due to more feedforward passes. LLMs are autoregressive decoders, so they generate one token at a time by using both input tokens and output tokens inferred earlier (Yenduri et al., 2023). Generating a text in a non-primary language requires more computational resources if the tokenization efficiency varies across supported languages, with some languages being segmented more effectively than others. Moreover, the current LLM price model requires paying for the number of both input and output tokens, which would make generation in any non-primary language more expensive for the end user. Thus, the cost of inference in this paper refers to the cost of executing the causal language modeling task (text generation), encompassing speed, context window usage, computational resources, and API call pricing.

This work proposes a way to benchmark tokenizers for a specific language, focusing on Ukrainian. We compare and analyze the current LLMs’ tokenization efficiency by conducting several experiments to check the efficiency for both general and domain-specific texts. We investigate the influence of grammatical and spelling errors on Ukrainian tokenization, explore the use of transliteration to improve results, and assess the effect of word form changes. Ukrainian tokenization is compared to the English language to determine the differences in their performance in terms of possible inference cost and context window filling. The approach we test for Ukrainian can be reproduced with a small amount of data for any other language. Even though the increased ratio of tokens per word is already a known conclusion for the Ukrainian language, we aim to provide a framework to benchmark current SOTA LLMs and future models not only in terms of their task performance but also in terms of their tokenization efficiency, as it directly affects the computational budget, speed of generation and API costs. We provide an in-depth check for language in general and specific domain corpora and examine the effect of mistakes in the text. The approach can be scaled to more domains, languages, or even dialects to ease the choice of the model for downstream tasks in low-resource or tokenizer secondary languages.

## 2 Related research

Tokenization efficiency was deeply researched for multilingual environments in general. Some papers focus on specific languages such as Thai and Hungarian (Csaki et al., 2023), Portuguese (Larcher et al., 2023), or French (Arnett et al., 2024), or just explore efficient ways to extend tokenizer vocabularies and embedding layers with new samples (Marchisio et al., 2023). However, even though the number of datasets and models skyrockets for the Ukrainian language (Saichyshyna et al., 2023), there is no in-depth research on the tokenization efficiency of current state-of-the-art language models for this language or a framework to benchmark the efficiency of tokenization in depth.

The difference in tokenization efficiency between different languages is a well-represented problem at this point (Petrov et al., 2023). The research presented in the paper “Language Model Tokenizers Introduce Unfairness Between Languages” provides a general comparison of multiple models like Llama, GPT-2 (Generative Pretrained Transformer), and RoBERTa (Robustly optimized Bidirectional Encoder Representations) in a set of diverse languages (Latin-based ones, the Germanic group, Cyrillic, Arabic, and multiple Asian and African groups).

It uses a metric called (Equations 1) to evaluate the performance of listed models in all those languages. It compares the computation required for training or inference, highlighting how the choice of tokenization schema differs from the optimal one.

(1) $$\text{tokenizer premiums}=\frac{\text{FLOP}s_{\text{estimated}}-\text{FLOP}s_{\text{optimal}}}{\text{FLOP}s_{\text{optimal}}}\;$$

Another way it can be calculated is by comparing target metric performance for the optimal and estimated (Equations 2), a difference in validation loss value between multiple models trained with different tokenization schemas.

(2) $$\text{tokenizer premiums}=\frac{\text{performanc}e_{\text{estimated}}-\text{performanc}e_{\text{optimal}}}{\text{performanc}e_{\text{optimal}}}$$

However, this metric would require an optimal baseline and training of multiple models based on different tokenizers, which can be time-consuming and computationally intensive. Additionally, some models and labs do not publish their tokenizers’ vocabularies and merge rules, making it impossible to reproduce their experiments (e.g., the Claude series). Such an approach would be efficient to compare small-scale open-source models, but it would be impossible to compare and evaluate models with a high parameter count and large vocabulary sizes due to the amount of data and computing necessary to run such experiments.

Equations 3 to compare the segmentation quality of tokenizers is tokenization fertility (Rust et al., 2021; Csaki et al., 2023). This metric shows the mean number of tokens necessary to encode a single word in a test dataset. This allows estimation of how many feedforward passes LLMs require to generate a word in a certain language on average and how many slots in the context window a word would take. Both these factors directly influence the speed and cost of inference and can make training of the model even more difficult. The token embeddings of LLM would not be meaningful on their own, so it increases the chance of mistakes during generation or misunderstandings of inputs.

(3) $$\text{tokenizer fertility}=\frac{\text{number of tokens}}{\text{number of words}}$$

It does not require training a model with an evaluated tokenizer, which makes the experiment and comparison faster and cheaper to conduct. The papers that use this metric only assessed the difference in segmentation between English and their target language. They do not research the tokenization efficiency for narrow domains, different styles, or topics, which affects the text segmentation as well. These experiments do not refer to the effects of syntax and grammar mistakes in the text, stylistic changes, or morphological transformations and their influence on the resulting token count. Tokenization fertility is used only to compare the bilingual tokenizer proposed by the authors with the original GPT-2.

A framework for evaluations of multilingual tokenizers was also proposed earlier, but it covers the influence of the tokenization schema on the performance of the model for a certain type of natural language processing task (Limisiewicz et al., 2023). Authors show how multilingual BPE tokenizers with overlapping parts of vocabularies can deteriorate the performance of the model on token-level tasks like part-of-speech tagging, token classification, or named entity recognition. However, such tokenizers prove to be efficient for sentence-level tasks such as text classification, semantic search retrieval, or reranking. Authors present a methodology and a code to measure tokenizers’ properties like vocabulary allocation average rank (a measure of distribution of tokens used to encode the target language), characters per token, and vocabulary overlap (the extent to which tokens get reused to represent multiple languages).

The idea behind characters per token is that longer tokens should store more information for a phonetic language. Phonetic language means that all words get read the same way as they are written, so there are no silent vowels. It does not need a more granular split into subwords to model the pronunciation. For example, the words “tough” and “though” in English, which is not a phonetic language, both use the subword “ough” (a tetragraph or a four-character sequence). It does not carry a meaning on its own and gets used as a building component, so it would be ok to have it as a separate token.

The framework itself proposes a model training approach, as the authors use multiple tokenizers to train small models to solve both token-level and sentence-level downstream tasks. Then they measure the correlation between the metrics of the tokenizer and the obtained task performance. The framework allows for choosing the most efficient tokenizer for the target language and domain, as no one tokenizer would work with the same efficiency for all of them. However, it focuses specifically on downstream tasks and ignores the effect of text style and domain.

In this research, we propose a simple framework to measure the tokenization efficiency of multilingual tokenizers across multiple domains, so developers can choose the best model and tokenizer for their target corpus in terms of tokens per word ratio. The presented methodology should provide quick evaluations on how the tokenizer’s efficiency in a low-resource language for a specific topic compares to the same one in the tokenizer’s primary language. The framework does not require model training and needs only a multilingual corpus and a general domain corpus. Thus, developers and researchers should be able to choose the model that uses the least amount of tokens to encode their data, save the cost of compute or API usage, and accelerate the generation by reducing the number of tokens per word.

The Ukrainian language is used to demonstrate the proposed approach, as it is a low-resource, morphologically rich language, resulting in a higher ratio of tokens per word compared to the Latin group of languages, and specifically English. This makes current SOTA LLMs less efficient for these languages, as it would require more compute for causal language modeling due to a higher level of text segmentation. So, by using this framework, developers and researchers can evaluate and choose the most efficient LLM for the Ukrainian language and their specific domain. The approach can be scaled to other languages, topics, and styles without training a new model.

## 3 Materials and methods

### 3.1 Datasets

The task of tokenization performance measurement requires us to have both a general language set and some specific domain-centered ones. We used the Brown corpora for the Ukrainian and English languages as examples of general texts to determine the baseline performance of multiple tokenizers in cases that do not require any uncommon or complex lexicon (Starko and Rysin, 2023). The NLTK implementation of Brown English corpora was loaded, so texts were merged by genres for experiments. This approach results in 15 larger texts for the English version instead of 500 separate ones, without affecting the calculations and measurements, as the research primarily focuses on the number of tokens per word in a text. Thus, concatenation by genres should not change the obtained values.

As for domain-specific texts, we used datasets presented in our previous research, which contain English and Ukrainian versions of Ukrainian laws, scientific article abstracts, and technical documentation (Maksymenko et al., 2023). The laws dataset is a sample of sentences from laws published on the Ukrainian Parliament website (both in English and Ukrainian). The scientific texts dataset contains abstracts from scientific articles mostly about economics and physics (Maksymenko et al., 2022). The technical documentation subset refers to the documentation of the VueJS framework, so it contains both natural language and code samples.

We used Grammarly’s Ukrainian grammatical errors corpus (GEC) and filtered for sentence pairs in which the incorrect and correct versions contained the same number of words. This was done to avoid token count fluctuations caused by differences in wording or complete rewrites (Syvokon et al., 2023). As a result, the majority of the retained corrections focused on word forms and spelling rather than sentence structure. This filtering process left us with 443 texts for analysis. The number of words in both versions was counted using the NLTK word tokenizer. If the word counts matched, the text was included in the benchmark. Otherwise, it was excluded, as such corrections involved significant rewrites.

For example, the dataset contains some stylistic errors, which are fixed by a complete rewrite of the sentence. This way, a count of tokens can differ significantly between versions, but it would not be representative, as the words and structure are completely different. We aimed to isolate the effect of common spelling and grammar case-matching mistakes, rather than comparing how a stylistic rewrite would affect tokenization.

More details on the dataset are provided in Table 1.

**Table 1:** Datasets statistics.

Dataset	Texts count	Bytes count	Characters count	Bytes per character	Words count	Characters per word	Sentence count	Words per text
Ukrainian Brown Corpus	1,420	13,978,170	7,794,197	1.79	1,373,811	5.67	69,018	967.47
English Brown Corpus	15	6,003,981	6,003,981	1.00	1,173,714	5.12	53,266	78,247.60
Ukrainian Laws (Ukrainian)	3,970	1,690,976	912,858	1.85	120,883	7.55	3,971	30.44
Ukrainian Laws (English)	3,970	989,529	989,509	1.00	162,156	6.10	3,971	40.84
Abstracts (Ukrainian)	3,910	3,096,762	1,679,202	1.84	240,761	6.97	7,608	61.56
Abstracts (English)	3,910	1,734,407	1,732,268	1.00	294,355	5.88	8,109	75.26
Code Documentation (Ukrainian)	7	141,577	94,185	1.50	17,688	5.33	450	2,524.00
Code Documentation (English)	7	90,751	90,731	1.00	18,560	4.89	458	2,651.43
GEC (corrected)	443	687,947	383,268	1.79	74,200	5.16	5,036	167.49
GEC (with mistakes)	443	685,471	383,271	1.79	74,200	5.16	5,021	167.49

### 3.2 Model selection and evaluation metrics

The following models were chosen for experiments:

- GPT-2: a baseline tokenizer to compare how much the efficiency of tokenization has changed since its release in 2019 (Budzianowski and Vulić, 2019);
- Ukrainian GPT-2. A GPT-2 with a custom tokenizer trained from scratch on Wikimedia dumps and the OSCAR dataset (Malteos/Gpt2-UK Hugging Face, 2024; Suárez et al., 2020). This model is added to show the primary language bias but reversed for English and Ukrainian, as this model was specifically created to encode and generate Ukrainian text without multilingual capabilities. Moreover, it allows for comparison of multilingual tokenizers of current SOTA LLMs to this specified model, which is trained for one low-resource language only.
- Llama 2 and Llama 3.1. 2 generations of LLMs by Meta, which use different vocabularies in their respective tokenizers, which allows for tracking changes in efficiency between multiple releases by the same research team (Touvron et al., 2023; Dubey et al., 2024).
- Mistral 7B/Large/Mixtral. A series of language models by Mistral Labs. These models have the same tokenizer, so they do not require separate measurements (Jiang et al., 2023, 2024).
- Mistral Nemo: a 12B model from the Mistral AI laboratory, which uses a different tokenizer from previous versions and was specifically designed for multilingual usage (Mistral AI Team, 2024).
- Gemma/Gemma 2/Gemini 1.5: 2 families of models from Google, which share the same tokenizer. Gemma is an open-source text-only model, while Gemini is a closed one and provides multidomain capabilities. These models use the same vocabulary. (Team et al., 2024; Team et al., 2024; Team et al., 2023).
- Qwen 1.5/2 VL: Both versions share the same tokenizer, and Qwen 2 is one of the few open-source multimodal LLMs, so it is important to evaluate its effectiveness for the Ukrainian language. Both versions use the same tokenizer vocabulary (Yang et al., 2024).
- Phi 2 and 3.5: open-source models by Microsoft. We chose both versions to monitor changes in tokenization efficiency across their generations, as they use different tokenizers (Gunasekar et al., 2023; Abdin et al., 2024).
- Mamba: a state-space model that proposes a different approach to attention for language modeling, which should make inference cheaper and provide higher stability for processing long context windows (Gu and Dao, 2023).
- Claude 3: an approximated version of the Claude tokenizer by Xenova Labs, as Anthropic does not provide direct access to their tokenizers (Kevian et al., 2024; Xenova/Claude-Tokenizer Hugging Face 2024, 2024).
- GPT-3.5/4: previous versions of OpenAI’s LLMs, which use the same tokenizer (OpenAI et al., 2023).
- GPT-4o/4o-mini: models by OpenAI with multimodal capabilities and a new tokenizer compared to the GPT-3.5/4 series.

The research specifically measures only the performance of tokenizers of the listed models, so no hyperparameter setups are required to replicate results.

For each tokenizer, the vocabulary size and the number of English tokens were recorded. Vocabulary size can be retrieved from the tokenizer implementation (for example, the vocab_size attribute if the tokenizer is loaded with the huggingface tokenizers package). The count of English tokens was retrieved by using the following rule: the token would be considered English if it contains only English (Latin) letters and a punctuation sign or underscores (punctuation signs and underscores cannot be the only content of the token, so it also has to contain at least one English character to be considered English). The methodology involves iterating over every token in the tokenizer’s vocabulary and checking its content according to the previously defined rule.

We also aimed to estimate the number of Ukrainian tokens, but some Cyrillic letter combinations can be present in multiple languages at the same time (for example, they can occur in both Ukrainian and Russian or contain valid Ukrainian characters, but the combination is more common in Russian). So, we measured all Cyrillic tokens that do not contain any specific symbols that are not present in the Ukrainian language. This measurement is approximated, as some subwords might not contain non-existent Ukrainian alphabet characters, but they are more common in other languages of the group. Such tokens can be filtered only manually, so “count of Cyrillic tokens” would be a more appropriate name for the value we calculated than “number of Ukrainian tokens.” This means that the real number of Ukrainian-only tokens is probably a bit less than the provided Cyrillic token count.

The token is considered Cyrillic if it contains only underscores or characters from the Cyrillic alphabet except for the non-Ukrainian symbols ё, ы, э, ъ, џ, Њ, љ, ћ, ј, ѕ, ќ, ѓ, and ў. These characters are not present in the Ukrainian alphabet, so if they appear in the token, this token would not be of interest for our research, as it gets used to encode Russian, Belarusian, Cyrillic Serbian, Bulgarian, etc. Current tokenizers use UTF-8 encoding, so Cyrillic symbols can be expressed as 2 bytes even if the tokenizer does not contain the symbol in its vocabulary at all (Tokenizers Encoding, 2025).

We propose to use metrics such as characters per token (CPT) and bytes per token (BPT) to check how meaningful English and Cyrillic tokens in the presented vocabularies are (Limisiewicz et al., 2023). The shorter the subword is, the less meaningful it is for a phonetic language. The subwords would become more ambiguous as they do not represent any reusable morpheme. It makes the model training harder, as it has to learn a more complex token embedding, which would encode multiple meanings at the same time. This way, CPT captures the semantic richness of a token for phonetic language and is a valid metric to evaluate tokenizers for any language of this group.

Tokenization fertility is the primary metric for our experiments and comparisons. It should be clarified that no preprocessing was used during the fertility calculation, so no stop words were removed, the letter case was left as it was, and no lemmatization or stemming technique was used. Furthermore, words were not tokenized individually, and tokenization was left as it would have been in a real case of a language modeling task.

This metric would be used as the main one to define the efficiency of tokenization for chosen models, as it represents the ratio between the number of tokens and words, which is easy to explain and interpret. The best possible value of tokenization fertility would be 1.0, as it would mean that the model uses one token per 1 word. This value is a global minimum for this metric. Such tokenization is impossible to achieve, as it would require including all words with all possible affix combinations into the tokenizer’s vocabulary. So, there are two main objectives for the tokenization of fertility:

- Minimize the value in general and make it as close to 1 as possible and
- Maintain consistent values across different languages and text domains (styles and topics) for a single tokenizer.

**Figure 1:**
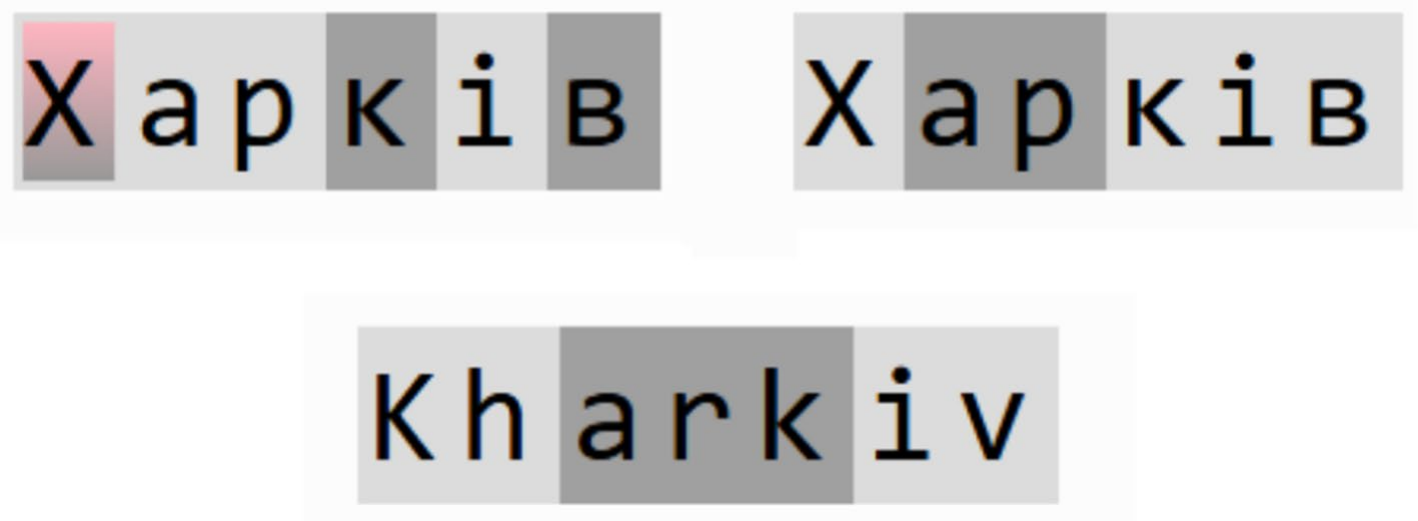
Example of tokenization of words in two languages.

Some languages or areas can use long and complex words with multiple roots or affixes, and it would be normal to tokenize them with multiple tokens. Even if a simple word gets encoded into numerous tokens, and this number is consistent across various languages, it would be an efficient option, as it does not introduce a bias toward one specific language. Figure 1 illustrates an example of this.

It showed the word “Харків”/“Kharkiv,” the name of a city in eastern Ukraine, and how GPT-3.5 and GPT-4o tokenizers segment it in two languages. Tokens are marked by a highlight color, where a change of tones of gray would mean that highlighted parts correspond to different tokens, and the red color indicates that a character gets encoded as a byte sequence (2 bytes in the case of Ukrainian due to the Unicode specification). Both tokenizers need three tokens in English to encode this word. GPT-4o requires three tokens to encode in Ukrainian, consistent with English. At the same time, GPT-3.5 does not contain the uppercase letter “Х” in its vocabulary at all, so it needs six tokens to encode the word, where two tokens are used to encapsulate just a single character. This way, GPT-3.5 would generate this single word longer than GPT-4o in Ukrainian due to inefficient tokenization (six feedforward passes vs. three ones).

Common language modeling metrics like perplexity, self-BLEU, ROUGE, and Distinct-N evaluate the model output and the quality of text generation. They do not evaluate the performance of the tokenizer used by the model. Their metrics focus on the alignment of the model’s outputs with human references. Such metrics do not reflect the efficiency of the tokenization process. Common language modeling metrics do not measure the quality of subword segmentation or its consistency across domains or word form changes. This way, traditional metrics listed earlier would not highlight the tokenizer impact specifically.

## 4 Experiment planning

We used the following approach to measure the efficiency of the listed tokenizers:

1. Measure the number of English and Cyrillic tokens in each tokenizer vocabulary. The same can be done for other languages too (for example, count the number of tokens, which can represent Chinese text). We used the methodology described in the previous section to determine whether a token is English, Cyrillic, or does not fall into any of these categories.
2. Measure CPT and BPT of those tokens to check the semantic richness of the tokens. The higher the metric value, the more meaningful the tokens are. This would work for phonetic and phonemic languages but can be replaced with a token per word for logographic ones.
3. Check how many tokens are necessary to tokenize a basic lower-and uppercase alphabet and some special symbols widely used in the target language. In our case, we added the apostrophe sign, which is a common element of Ukrainian words, to the test. This gives 66 characters in total, which we tokenize individually (33 lowercase letters and 32 uppercase without “ь” and apostrophes). It provides information on how many times a tokenizer falls back to a byte representation to describe a single letter. Such a representation would require LLM to execute two feedforward passes to generate just one letter, as each Ukrainian letter is encoded with 2 bytes in UTF-8. This is even more crucial for Asian or Arabic languages, which can use up to 4 bytes per character.
4. Measure the performance of tokenizers on English and Ukrainian Brown corpus versions to determine the degree of bias of the tokenizer toward the English language in comparison to Ukrainian. Brown corpora were designed to be balanced between multiple genres, styles, and purposes of text. Brown can be replaced with any general domain text corpus for the target language. We recorded the total token count, average number of tokens per text, and tokenization fertility. The obtained values will be used as baselines for further experiments.
5. The next step is to measure the same set of metrics on specialized corpora (laws, code documentation, and scientific articles in our case). We compare these values to the results obtained on the Brown corpus to find out how much the tokenization fertility drops for a narrower, domain-specific dataset. The step can be reproduced for any other domains and does not require a large number of texts. This can even be extended to measuring the performance on specific regional dialects.
6. Then, we compare the efficiency between correct and incorrect spelling. We measure a total token count and tokenization fertility for a version of text with mistakes and a corrected version. As it was stated earlier, the dataset was filtered specifically to leave only pairs with the same number of words in both versions, so no style corrections or significant rewrites should affect the experiment. Word fusions were not tested in the current experiments. Common chatbot systems have to deal with incorrect grammar or spelling constantly, so it is important to measure whether the tokenization fidelity would change significantly due to an error in the input. For our experiment, we used the Grammarly GEC dataset for the Ukrainian language.
7. Another aspect we wanted to check is the effect of transliteration on tokenization efficiency. Models have significantly more English tokens, so there is a possibility that those subwords can cover Ukrainian letter combinations too, which can at least save space in the context window, as generating a transliterated output would be too unstable and difficult to decode back into Cyrillic text (LLMs do not follow transliteration rules strictly during a generation). Transliteration is fast and does not require an additional model in the inference pipeline, so if it gives an improvement for tokenization, it would be worth exploring further. We used a standard transliteration proposed by the Ukrainian government and measured fertility on the same Ukrainian Brown corpus. This approach can be checked for any non-Latin language, as common tokenizers are heavily skewed toward subwords of Latin languages. We would like to investigate it further in terms of LLM performance for question-answering tasks or pretraining from scratch on transliterated corpora.
8. Finally, we measure the tokenization fertility for multiple grammar cases. The Ukrainian language has seven cases. We fetched 19,000 words to measure how much tokenization differs between the nominative case and others on average. The approach is universal and can be reproduced for any other morphologically rich language.

It is important to note that CPT and BPT would not be as useful for benchmarking of logographic languages like Chinese (languages where a character can represent an entire concept rather than a sound). Characters that are already informationally dense and can stand for a whole word in non-logographic ones. A low CPT value does not necessarily indicate lower tokenizer efficiency. It would be more useful to check the number of tokens per word for such languages to determine how often a tokenizer falls back to a byte representation.

These metrics would still be useful for phonemic languages such as English, Korean, Turkish, and others (ones where writing and letter combinations represent distinct sounds instead of a direct pronunciation). Longer tokens would still capture more meaning, but it would require a large vocabulary dedicated to this specific language to capture all variations.

## 5 Results

### 5.1 Vocabulary metrics

First, we measured the number of Latin and Cyrillic tokens in the listed tokenizers, as specified in the experiment planning. All characteristics of the models’ tokenizers are presented in Table 2 (vocabulary size, the count of Latin (English) and Cyrillic tokens, and the mean and median CPT and BPT for both groups of tokens).

**Table 2:** Characteristics of the chosen tokenizers.

Model	Vocabulary Size	English					Cyrillic				
		Token count	Mean CPT	Median CPT	Mean BPT	Median BPT	Token count	Mean CPT	Median CPT	Mean BPT	Median BPT
GPT-2	50,257	14,829	4.81	4	4.81	4	16	1.00	1	2.00	2
Ukrainian GPT-2	50,304	1,720	3.38	3	3.38	3	11,248	4.68	4	9.36	8
Llama 2	32,000	24,084	5.65	5	6.81	7	2,768	4.11	4	8.71	8
Llama 3.1	128,262	28,158	5.49	5	5.49	5	2,558	3.61	3	7.21	6
Mistral 7B/Large/ Mixtral	32,768	25,019	5.76	5	6.92	7	1,625	3.58	3	7.59	7
Mistral Nemo	131,072	22,447	4.92	5	4.92	5	2,735	3.56	3	7.12	6
Gemma/Gemma 2/Gemini	256,000	168,995	6.64	6	7.79	8	12,009	5.20	5	10.94	10
Qwen 1.5/2 VL	151,657	27,376	5.52	5	5.52	5	1,791	3.48	3	6.96	6
Phi 2	50,295	14,829	4.81	4	4.81	4	16	1.00	1	2.00	2
Phi 3.5	32,011	24,084	5.65	5	6.81	7	2,768	4.11	4	8.71	8
Mamba	50,277	16,431	4.88	4	4.88	4	259	2.23	2	4.46	4
Claude 3 (approximated)	65,000	27,102	5.51	5	5.51	5	293	2.51	2	5.01	4
GPT-3.5/4	100,263	27,329	5.52	5	5.52	5	435	2.87	3	5.73	6
GPT-4o/4o-mini	200,000	37,839	5.11	5	5.11	5	4,660	3.70	3	7.40	6

As can be seen in Table 2, English tokens consist of 4–5 characters on average, whereas Cyrillic tokens mostly contain 3–4 characters per token. Tokenization would be even harder for the Ukrainian language due to its morphological richness (many word forms for the same root, case markings, and multiple affixes can be added to a word to completely change its meaning). The best result in terms of the CPT metric for Ukrainian is obtained with Gemma/Gemini (five characters per token).

A significant increase in vocabulary sizes for models released in 2024 has to be noted. For example, GPT-3.5/4 has only 435 Cyrillic tokens and a general vocabulary size of 100,235 tokens. GPT-4o and 4o-mini have increased vocabulary by 2 times (200,000 tokens) and have 10 times more Cyrillic tokens (4,660). Models like Gemma and GPT-4o achieve the best results by CPT specifically due to their large vocabularies, as they can cover more subwords and their combinations. This allows for more meaningful tokenization of non-Latin texts, rather than segmenting them into small chunks without rich semantic value.

The same cannot be said about Llama models. Llama 3 achieved a vocabulary 4 times larger than Llama 2, but the number of Cyrillic tokens and CPT decreased for it.

### 5.2 Alphabet knowledge test

As stated in Section 4 (Experiment planning), the first step is to measure the tokenization of the basic alphabet. In this experiment, we measure the number of tokens for each letter of the target language alphabet (Ukrainian for the current research). This test detects whether all letters are represented in the tokenizer’s vocabulary and checks if the use of some rare symbols leads to the byte fallback (representing a character using byte Unicode encoding). The number of tokens would be the same as the number of bytes necessary to represent the character in the Unicode table so that it can take up to 4 tokens per letter in the worst case.

Table 3 shows the result of the alphabet knowledge experiment. Measurements shown in the table indicate that Phi 2 and vanilla GPT-2 perform the worst in Ukrainian alphabet tokenization, utilizing bytes for 74% of Ukrainian characters. Models like GPT-4o and Llama 3.1 contain all Ukrainian symbols in their vocabularies except for the capital “ґ.” The best results are obtained by the Ukrainian GPT, Qwen, and the Gemma/Gemini family, as they do not use bytes at all to tokenize Ukrainian text. So, Gemini gets the best result among closed models, and Gemma and Qwen are the only open-source large models (2B+) that cover all symbols.

**Table 3:** Ukrainian alphabet knowledge check.

Model	Number of known characters	Number of unknown characters	Tokenizer fertility
GPT-2	17	49	1.74
**Ukrainian GPT-2**	**66**	**0**	**1**
Llama 2	61	5	1.08
Llama 3.1	65	1	1.02
Mistral 7B/Large/Mixtral	59	7	1.11
Mistral Nemo	62	4	1.07
**Gemma/Gemma 2/Gemini**	**66**	**0**	**1**
**Qwen 1.5/2 VL**	**66**	**0**	**1**
Phi 2	17	49	1.74
Phi 3.5	61	5	1.08
Mamba	49	17	1.26
Claude 3 (approximated)	55	11	1.17
GPT-3.5/4	53	13	1.20
GPT-4o/4o-mini	65	1	1.02

Models with the most Ukrainian alphabet in the tokenization marked bold.

### 5.3 Tokenization fertility measurement in the general domain

The next step is to measure all tokenizers with the English and Ukrainian Brown corpora to find out how well they segment general domain texts in both languages (Table 4).

**Table 4:** Brown corpora tokenization fertility measurements.

Model	English			Ukrainian		
	Total number of tokens	Average number of tokens per text	Tokenizer fertility	Total number of tokens	Average number of tokens per text	Tokenizer fertility
GPT-2	1,267,946	84,529.73	1.08	8,667,064	6,103.57	6.31
**Ukrainian GPT-2**	2,294,904	152,993.60	1.96	**1,779,807**	**1253.39**	**1.30**
Llama 2	1,431,009	95,400.60	1.22	3,152,299	2,219.93	2.29
**Llama 3.1**	1,268,917	84,594.47	1.08	**2,578,473**	**1,815.83**	**1.88**
Mistral 7B/Large/Mixtral	1,382,732	92,182.13	1.18	3,404,781	2,397.73	2.48
Mistral Nemo	1,287,915	85,861.00	1.10	2,808,059	1,977.51	2.04
**Gemma/Gemma 2/Gemini**	**1,256,270**	**83,751.33**	**1.07**	2,640,770	1,859.70	1.92
Qwen 1.5/2 VL	1,279,865	85,324.33	1.09	3,882,103	2,733.88	2.83
Phi 2	1,267,946	84,529.73	1.08	8,666,816	6,103.39	6.31
Phi 3.5	1,431,009	95,400.60	1.22	3,152,299	2,219.93	2.29
Mamba	1,279,625	85,308.33	1.09	4,606,862	3,244.27	3.35
Claude 3 (approximated)	1,285,667	85,711.13	1.10	4,742,885	3,340.06	3.45
GPT-3.5/4	1,269,116	84,607.73	1.08	4,560,959	3,211.94	3.32
GPT-4o/4o-mini	1,256,484	83,765.60	1.07	2,719,430	1,915.09	1.98

Models with the least amount of tokenization per Ukrainian text marked bold.

Vanilla GPT-2 and Phi 2 perform the worst in terms of Ukrainian tokenization, rendering them unfit for Ukrainian language modeling (with over 6 + tokens per word). This poor performance was consistently observed across other experiments as well, as reflected in the measurement tables. Therefore, we will focus on other models in subsequent sections. As expected, GPT-2, which was specifically trained for the Ukrainian language, achieves the best fertility score (just 1.30). However, this model lacks multilingual capabilities, as its performance drops significantly on the English corpus (1.96 compared to 1.22—the worst case among other models). Despite not using bytes during Ukrainian text tokenization, the Qwen tokenizer yields one of the poorest results among open-source models, with a fertility value of 2.89. In contrast, Llama 3.1 delivers the highest tokenization quality for the Ukrainian Brown corpus, with Gemma/Gemini following closely behind.

Llama 3.1 outperforms 2.0, but as was mentioned earlier, the CPT and count of Cyrillic tokens are lower for the third version. It may indicate that the Llama 3 model family tokenizer was trained with more Ukrainian texts than other languages that use a Cyrillic alphabet. It is impossible to prove without detailed training dataset research (which is not published publicly), but we can only suppose that this is the reason for overperformance in comparison to other models. This is only a speculative assumption, as the training data of this tokenizer is not open-sourced, and we can only measure its efficiency and approximate number of tokens, which can be used to segment Ukrainian texts.

We can see the same behavior with Ukrainian GPT-2, which has 50,304 tokens in vocabulary but outperforms every other tokenizer by a fertility value. It was trained specifically with Ukrainian as a primary language, and the vocabulary contains mostly tokens to segment Ukrainian text. So, if Llama 3.1 had mainly Ukrainian texts to represent the Cyrillic group during tokenizer training, it would explain why it gives better results than other foundational models’ tokenizers.

It is worth noting that closed models like Claude and GPT-3.5/4 look significantly less efficient for the Ukrainian corpus than other models (even smaller open-source ones), as they tend to use 3 + tokens per word even for general texts. Furthermore, there is a clear improvement in terms of Ukrainian tokenizer fertility between model generations, as all of them get much higher tokenization efficiency with new versions. It is primarily achieved with the increased vocabulary size (usually around +100,000 tokens in newer versions), which can be seen in Table 2. However, Phi 3.5 is the only model that has a smaller vocabulary than its predecessor but gets better benchmark results. This model is closer to previous Mistral and Llama versions, both in terms of vocabulary size and fertility, so we can conclude that while Phi 2 was mostly monolingual, Phi 3 is trained on a more diverse dataset. Its tokenizer is on the same level as earlier models by Meta and Mistral for Ukrainian tasks.

### 5.4 Tokenization of domain-specific texts for fertility measurement

The next step is to evaluate these models using domain-specific texts: laws, scientific articles, abstracts, and code documentation. We used the Brown corpora measurements as a baseline tokenization fertility value to assess the level of performance degradation (how much the tokenization fertility would increase) on more narrowly specialized tasks (Table 5).

**Table 5:** Narrowly specialized corpora tokenization fertility measurements.

Model	English				Ukrainian			
	Brown corpus fertility	Laws fertility	Scientific fertility	Code documentation fertility	Brown corpus fertility	Laws fertility	Scientific fertility	Code documentation fertility
GPT-2	1.08	1.06	1.17	1.54	6.31	8.75	7.99	4.65
Ukrainian GPT-2	1.96	2.07	2.17	2.16	1.30	1.17	1.45	1.79
Llama 2	1.22	1.26	1.36	1.51	2.29	2.76	2.71	2.14
**Llama 3.1**	1.08	1.10	1.15	1.25	**1.88**	**2.00**	**2.23**	**1.69**
Mistral 7B/Large/ Mixtral	1.18	1.24	1.31	1.53	2.48	3.00	2.94	2.28
Mistral Nemo	1.10	1.16	1.14	1.28	2.04	2.45	2.45	1.81
Gemma/Gemma 2/Gemini	1.07	1.12	1.12	1.35	1.92	2.28	2.23	1.80
Qwen 1.5/2 VL	1.09	1.14	1.17	1.26	2.83	3.64	3.40	2.29
Phi 2	1.08	1.06	1.17	1.50	6.31	8.74	7.99	4.62
Phi 3.5	1.22	1.26	1.36	1.51	2.29	2.76	2.71	2.14
Mamba	1.09	1.07	1.14	1.44	3.35	4.26	4.07	2.78
Claude 3 (approximated)	1.10	1.07	1.16	1.39	3.45	4.46	4.17	2.74
GPT-3.5/4	1.08	1.10	1.15	1.25	3.32	4.40	4.02	2.56
GPT-4o/4o-mini	1.07	1.10	1.14	1.25	1.98	2.29	2.35	1.74

Model with minimal tokenization fertility marked bold.

Results show that even though there is a performance degradation for English texts, it is not as significant as for Ukrainian ones. The biggest fertility score deterioration happens with code documentation in the English language, which can be explained by the structure and style of the code inclusions (more line breaks, formatting, and tabulation). For laws and abstracts, performance changes by 0.14 at worst (Llama 2 Brown vs. scientific), so domain-specific words and phrases can be considered less influential for the English language due to vocabulary size, which covers lots of possible subwords.

However, the domain significantly influences the Ukrainian language performance, as most models get worse by almost half a token on average for laws and scientific texts. Closed models such as GPT-3.5/4 and Claude can reach up to 4.4 tokens per word when they are tasked with narrow topics. The least performance degradation is observed for Llama 3.1 with Gemma/Gemini and GPT 4o/4o-mini, which can be attributed to their significantly larger vocabularies.

The code documentation earns better fertility scores compared to general texts or other domains because LLMs are pre-trained for coding tasks and receive numerous code samples during both the tokenizer tuning and pretraining stages. The results are still significantly worse than English ones (the difference can be as high as one token per word). However, large tokenizers outperform a smaller monolingual tokenizer of Ukrainian GPT-2. It can be explained by the training set composition and quality of data in terms of diversity and coverage of different domains and tasks, including programming. Moreover, this tokenizer is two times smaller than the vocabulary of Llama 3.1, four times smaller than GPT-4o’s vocabulary, and five times smaller than Gemini’s. Such a size allows those models to keep a separate set of tokens for coding tasks specifically (such as tabulations, space sequences, operator combinations, and other specific sets of characters). They can use their high number of English tokens to encode variable, function, class, and method names better than the monolingual tokenizer for a low-resource language.

The word length increases in specialized corpora for both English and Ukrainian, as shown in Table 1. However, English vocabulary has more subwords to cover these words without oversegmentation (splitting words into letter or byte sequences). A similar behavior can be seen for models with larger Ukrainian vocabularies. Tokenizers with smaller vocabularies are significantly more affected as their tokenization fertility rates increase by one or more tokens on average. This suggests that as long as BPE-like tokenizers are the standard solution for language models, the increase of their vocabulary size is the only way to keep them consistent across multilingual and multidomain tasks. The only other viable solution for the BPE approach is the creation of a monolingual tokenizer and model (like Ukrainian GPT-2), which does not have to share vocabulary between multiple languages. More space would be used for less frequent subwords, allowing for better coverage of narrow topics, styles, and language subdomains.

### 5.5 Influence of mistakes on tokenization fertility

The next analysis examines how mistakes impact tokenization quality. Table 6 provides the full results for this experiment (including token counts for both correct and incorrect text versions and their tokenization fertility values).

**Table 6:** Influence of Ukrainian grammatical errors on tokenization fertility.

Model	Token count for incorrect versions	Fertility for incorrect versions	Token count for correct versions	Fertility for correct versions
GPT-2	430,029	5.796	430,301	5.799
Ukrainian GPT-2	96,108	1.295	95,948	1.293
Llama 2	161,522	2.177	161,441	2.176
Llama 3.1	133,584	1.800	133,485	1.799
Mistral 7B/Large/ Mixtral	172,183	2.321	172,113	2.320
Mistral Nemo	143,577	1.935	143,480	1.934
Gemma/Gemma 2/Gemini	134,580	1.814	134,521	1.813
Qwen 1.5/2 VL	193,002	2.601	193,114	2.603
Phi 2	429,924	5.794	430,223	5.798
Phi 3.5	161,522	2.177	161,441	2.176
Mamba	228,140	3.075	228,295	3.077
Claude 3 (approximated)	236,612	3.189	236,836	3.192
GPT-3.5/4	225,144	3.034	225,317	3.037
GPT-4o/4o-mini	139,026	1.874	138,978	1.873

The difference in tokenization is insignificant, and changes in fertility score become visible only at the third sign after the comma, so the influence of incorrect spelling or word forms can be considered low for all listed models. The tokenization process is deterministic, so it is not influenced by random state or any other factor except for the tokenizer itself and the input text. However, using correct variants yields slightly better results, but this improvement is too small to affect the cost of inference or drastically reduce free slots in the context window.

We plan to extend this experiment with more benchmark texts in our further research (both Ukrainian and other languages) to measure the effect of grammatical and spelling errors on the tokenization fertility of current foundation language models. Moreover, at this stage, we did not take word fusions (lack of space) into account, so this should also be checked in further experiments. An additional cause underlying the low influence of mistakes is the low Levenshtein distance between correct and incorrect versions of texts. Minimal value is 1, maximum is 107, and median is 9. 90% of versions have a distance under 27 characters. The dataset needs to be expanded to include more error types and additional samples in future research to achieve more accurate results.

### 5.6 Tokenization fertility measurement with Latin transliteration

Transliteration preprocessing was measured, and the results are presented in Table 7. It improves the tokenization fertility only for those models that previously had fertility scores higher than 2.5. Newer models like Llama 3.1, GPT-4o, 4o-mini, Gemma, and Gemini with large vocabularies tend to perform better with raw Ukrainian text without any transformation. With a growth in vocabulary size and improvements in tokenizer training sets (increased quantity and quality of multilingual samples), the transliteration approach becomes inefficient and unnecessary.

**Table 7:** Latin transliteration effect on tokenization fertility for the Ukrainian language.

Model	English Brown corpus fertility	Ukrainian Cyrillic Brown corpus fertility	Ukrainian Latin Brown corpus fertility
GPT-2	1.08	6.31	2.74
Ukrainian GPT-2	1.96	1.30	3.16
Llama 2	1.22	2.29	2.65
Llama 3.1	1.08	1.88	2.40
Mistral 7B/Large/Mixtral	1.18	2.48	2.72
Mistral Nemo	1.10	2.04	2.36
Gemma/Gemma 2/Gemini	1.07	1.92	2.28
Qwen 1.5/2 VL	1.09	2.83	2.50
Phi 2	1.08	6.31	2.74
Phi 3.5	1.22	2.29	2.65
Mamba	1.09	3.35	2.63
Claude 3 (approximated)	1.10	3.45	2.63
GPT-3.5/4	1.08	3.32	2.49
GPT-4o/4o-mini	1.07	1.98	2.26

However, it allows us to decrease the fertility rate of the vanilla GPT-2 and Phi 2 down to values obtained from models like Llama 2 on raw Ukrainian text. The result is even better than raw Ukrainian tokenization by models like GPT-3.5/4 and Claude 3. They would still require significant fine-tuning for transliterated Ukrainian to understand and use the language, but tokenizers and initial weights of models can be reused.

We plan to research this approach further in our future studies for smaller models. Transliteration improved tokenization fertility only in models that already had large vocabularies (100,000 + tokens), so it would be more reasonable to apply it for small models trained from scratch, as it decreases the number of token embeddings to teach.

### 5.7 Grammar case tokenization

The final experiment was to measure the change in tokenization fertility as the grammatical case of input words is changed. Morphologically rich languages like Ukrainian are characterized by grammar case matching and different forms for quantity, tense, or gender. Table 8 presents the results of the grammar case tokenization test, measuring tokenization fertility for each of the 7 cases and the mean difference from the nominative case (the infinitive of a word in Ukrainian).

**Table 8:** Tokenization fertility by grammatical cases.

Model	Nominative	Genitive	Dative	Accusative	Instrumental	Locative	Vocative	Mean difference from nominative
GPT-2	12.24	12.63	13.85	13.26	12.41	13.51	12.51	0.787
Ukrainian GPT-2	**3.46**	**3.57**	**3.85**	**3.79**	**3.53**	**3.86**	**3.74**	**0.266**
Llama 2	4.68	4.76	5.27	5.13	4.74	5.16	4.83	0.304
Llama 3.1	4.46	4.59	4.98	4.88	4.55	4.90	4.71	0.304
Mistral 7B/Large/Mixtral	4.95	5.01	5.57	5.48	4.99	5.54	5.07	0.327
Mistral Nemo	**4.39**	**4.51**	**4.95**	**4.86**	**4.46**	**4.82**	**4.57**	**0.299**
Gemma/Gemma 2/Gemini	4.01	4.12	4.64	4.47	4.11	4.55	4.20	0.339
Qwen 1.5/2 VL	5.39	5.54	6.04	6.05	5.44	6.34	5.55	0.438
Phi 2	12.24	12.63	13.85	13.26	12.41	13.51	12.51	0.787
Phi 3.5	4.68	4.76	5.27	5.13	4.74	5.16	4.83	0.304
Mamba	6.53	6.64	7.30	7.20	6.51	7.39	6.71	0.433
Claude 3 (approximated)	6.64	6.65	7.25	7.27	6.54	7.36	6.81	0.373
GPT-3.5/4	6.51	6.65	7.35	7.24	6.48	7.47	6.80	0.495
GPT-4o/4o-mini	4.34	4.43	4.91	4.82	4.37	4.80	4.57	0.310

The best models for Ukrainian text marked bold.

Expectedly, the best result is achieved by a mostly monolingual Ukrainian GPT-2, as the mean difference between all cases and the nominative one is just 0.266. Mistral Nemo achieves the best result among all current multilingual LLMs, with a 0.299 difference. It is closely followed by both Llama models, Phi 3.5, and GPT-4o/4o-mini. It is worth noting that all models’ tokenization performance deteriorates with changing word forms, which again proves a higher difficulty with adapting subword tokenization for morphologically rich languages. It is not a problem of a specific tokenizer or model, as this disadvantage is a property of subword and BPE-like tokenizers in general.

## 6 Discussion

In the scope of this study, we presented a detailed and comprehensive analysis of the current state-of-the-art large language models’ tokenization performance for the Ukrainian language and compared it to English.

Results prove that all listed models deteriorate in tokenization performance on Ukrainian texts compared to their English counterparts. For the most recent models, this difference can reach up to 2.5 additional tokens per word. On average, the majority of models require one additional token per word to encode semantically equivalent content in Ukrainian. This observation is drawn from tokenization measurements conducted on both a general-domain corpus (the Brown corpus) and specialized corpora, including legal texts, scientific abstracts, and code documentation.

This increase in the number of tokens per word directly affects the speed of text generation, as the model would need at least an additional feedforward pass to generate a word on average. It makes not only the inference during a causal language modeling task (text generation) longer but also increases the necessary number of computational resources to create a single text and increases the cost for model users (in case of API usage). That is why the number of tokens per word (tokenization fertility) was used as a primary metric during our research. It enables the assessment of a generative language model for use in a specific domain or language prior to text generation and benchmarking on semantic tasks. The model can be inefficient from the start due to a significantly biased tokenizer, which makes it slower and more expensive for the user’s target language.

Gemini models seem to achieve the best tokenization fertility among all closed ones, likely due to their largest vocabulary size. It is closely followed by GPT-4o and 4o-mini, which show drastic improvements compared to GPT-3.5 and 4. Claude gave the worst results among all closed models, but we used an approximated version of its tokenizer, so we would like to remeasure it if Anthropic open-sources their tokenizer down the line.

Llama 3.1 has the best tokenization efficiency among open-source models, even though it has fewer Cyrillic tokens in its vocabulary than Gemma or even Llama 2. It is tied with Gemma/Gemma 2, but Llama 3.1 has higher benchmark scores, so we recommend it as currently the best open model for Ukrainian NLP tasks.

While Gemma/Gemini has the highest Cyrillic CPT and the highest number of Cyrillic tokens, it still loses to Llama 3.1 in terms of tokenization fertility. This can be explained by the large number of Russian tokens in Gemma’s vocabulary, which makes the Cyrillic token count higher, but they do not boost performance for the Ukrainian language. Those tokens do not contain any symbols specific to the Russian alphabet, but these letter combinations are not common in the Ukrainian language.

Mamba, the only non-attention model among those tested, delivers tokenization performance close to GPT-3.5/4, but in theory, its inference cost should be lower, making such tokenization potentially less harmful for Ukrainian modeling tasks compared to attention-based counterparts.

Grammatical errors turned out to be insignificant, even in the Ukrainian language. Transliteration preprocessing has become obsolete with the expansion of vocabularies and only worsens performance in the latest models. All models show noticeable deterioration when handling word grammatical case change, which again proves the ineffectiveness of subword tokenization for morphologically rich languages.

Finally, we have shown that tasks that require the usage or generation of narrow topic texts would decrease the effectiveness of tokenization even more for Ukrainian. However, it does not affect English as much. It can be explained by the fact that English is the primary language of their vocabulary and contains a large enough set of tokens to cover those rare words and forms. A smaller monolingual tokenizer outperforms large multilingual tokenizers even more for narrow domains and topics. However, it is expected to perform worse in domains that may use a mix of different languages, like technical documentation or coding tutorials.

Results show that tokenizers with a large vocabulary size (100,000 or more) tend to be less affected by the domain or language of the task. Thus, as long as BPE-like subword tokenization stays a standard solution for language modeling preprocessing, larger vocabularies are the only way to make the tokenization more consistent across languages and domains. The model would still have a bias toward the primary language, but the increase in tokenization fertility would not be as prominent (like with GPT-3.5, for which the number of tokens per word would grow 3 times for Ukrainian in comparison to English).

We plan to research embedding decoding approaches further to explore whether sentence embeddings can replace raw text, providing additional context to LLMs and eliminating the need for constant, inefficient tokenization in multilingual environments, particularly for the Ukrainian language.

Moreover, it was mentioned earlier that a transliteration can be an interesting way to continue this research for optimization of the parameter count of the model. Instead of training a large tokenizer and corresponding token embeddings, it can be more optimal for small models to be trained with a Latin-transliterated corpus and a small vocabulary, which would consist only of Latin-like/English subwords.

Another direction for further improvement and development of this study is to dive deeper into how mistakes influence tokenization efficiency by researching word fusions and stylistic or structural errors. The approach can be scaled to compare local dialects, checking how efficiently a tokenizer segments a dialect version of the language, rather than the more commonly used one.

The proposed framework can be reused for other languages, domains, or dialects and allows for comparing the efficiency of tokenization for the target language and topic to the primary language of the model and its tokenizer. This methodology should facilitate the selection of models for tasks, enabling evaluation not only by their performance on this specific task but also by the number of tokens per word. A high token-to-word ratio can result in longer and more expensive text generation in causal language modeling tasks and make generalization more difficult in natural language understanding due to poor segmentation quality.

## 7 Limitations

The main limitation of our approach is that it evaluates only the efficiency of word segmentation into tokens; it does not assess how well a model understands or uses language or certain domain-specific information in terms of grammar, knowledge, and style. Therefore, this method should be used only as one component in benchmarking a model for a specific language or domain. It can also be applied to evaluate the quality of a tokenizer’s vocabulary before training a model for multilingual use cases.

Another limitation applies to proprietary models developed by AI laboratories that do not open-source their tokenizers. Although tokenization fertility can still be measured using APIs—by passing test text as an input prompt and observing the number of input tokens—this approach increases testing costs and requires customizing the benchmarking pipeline for such models.

## Data Availability

The raw data supporting the conclusions of this article will be made available by the authors, without undue reservation.
